# Association between *CYP2D6* Genotypes and the Risk of Antidepressant Discontinuation, Dosage Modification and the Occurrence of Maternal Depression during Pregnancy

**DOI:** 10.3389/fphar.2017.00402

**Published:** 2017-07-17

**Authors:** Anick Bérard, Andrea Gaedigk, Odile Sheehy, Christina Chambers, Mark Roth, Pina Bozzo, Diana Johnson, Kelly Kao, Sharon Lavigne, Lori Wolfe, Dee Quinn, Kristen Dieter, Jin-Ping Zhao, the OTIS (MotherToBaby) Collaborative Research Committee

**Affiliations:** ^1^Faculty of Pharmacy, University of Montreal Montreal, QC, Canada; ^2^Research Center, CHU Sainte-Justine Montreal, QC, Canada; ^3^Division of Clinical Pharmacology, Toxicology and Therapeutic Innovation, Children's Mercy-Kansas City Kansas City, MO, United States; ^4^School of Medicine, University of Missouri-Kansas City Kansas City, MO, United States; ^5^Department of Pediatrics, University of California San Diego La Jolla, CA, United States; ^6^Pregnancy Risk Network, NYS Teratogen Information Service Binghamton, NY, United States; ^7^Motherisk Program, Hospital for Sick Children Toronto, ON, Canada; ^8^California Teratogen Information Service San Diego, CA, United States; ^9^Connecticut Pregnancy Exposure Information Service, Division of Human Genetics, University of Connecticut Health Center Farmington, CT, United States; ^10^Texas Teratogen Information Service, University of North Texas Denton, TX, United States; ^11^Arizona Pregnancy Riskline, Colleges of Medicine and Pharmacy, University of Arizona Tucson, AZ, United States; ^12^Illinois Teratology Information Service Chicago, IL, United States

**Keywords:** *CYP2D6* genotypes, antidepressant discontinuation, dosage modification, maternal depression in pregnancy

## Abstract

**Importance:** Polymorphic expression of drug metabolizing enzymes affects the metabolism of antidepressants, and thus can contribute to drug response and/or adverse events. Pregnancy itself can affect CYP2D6 activity with profound variations determined by *CYP2D6* genotype.

**Objective:** To investigate the association between *CYP2D6* genotype and the risk of antidepressant discontinuation, dosage modification, and the occurrence of maternal *CYP2D6*, Antidepressants, Depression during pregnancy.

**Setting:** Data from the Organization of Teratology Information Specialists (OTIS) Antidepressants in Pregnancy Cohort, 2006–2010, were used. Women were eligible if they were within 14 completed weeks of pregnancy at recruitment and exposed to an antidepressant or having any exposures considered non-teratogenic.

**Main Outcomes and Measures:** Gestational antidepressant usage was self-reported and defined as continuous/discontinued use, and non-use; dosage modification was further documented. Maternal depression and anxiety were measured every trimester using the telephone interviewer-administered Edinburgh Postnatal Depression Scale and the Beck Anxiety Inventory, respectively. Saliva samples were collected and used for *CYP2D6* genotype analyses. Logistic regression models were used to calculate crude and adjusted odds ratios (OR) with 95% confidence intervals.

**Results:** A total of 246 pregnant women were included in the study. The majority were normal metabolizers (NM, *n* = 204, 83%); 3.3% (*n* = 8) were ultrarapid metabolizers (UM), 5.7% (*n* = 14) poor metabolizers (PM), and 8.1% (*n* = 20) intermediate metabolizers (IM). Among study subjects, 139 women were treated with antidepressants at the beginning of pregnancy, and 21 antidepressant users (15%) discontinued therapy during pregnancy. Adjusting for depressive symptoms, and other potential confounders, the risk of discontinuing antidepressants during pregnancy was nearly four times higher in slow metabolizers (poor or intermediate metabolizers) compared to those with a faster metabolism rate (normal or ultrarapid metabolizers), aOR = 3.57 (95% CI: 1.15-11.11). Predicted CYP2D6 metabolizer status did not impact dosage modifications. Compared with slow metabolizers, significantly higher proportion of women in the fast metabolizer group had depressive symptom in the first trimester (19.81 vs. 5.88%, *P* = 0.049). Almost 21% of treated women remained depressed during pregnancy (14.4% NM-UM; 6.1% PM-IM).

**Conclusions and Relevance:** Prior knowledge of *CYP2D6* genotype may help to identify pregnant women at greater risk of antidepressant discontinuation. Twenty percent of women exposed to antidepressants during pregnancy remained depressed, indicating an urgent need for personalized treatment of depression during pregnancy.

## Key points

**Question:** Do women with certain *CYP2D6* genotypes have higher risk of antidepressant discontinuation, dosage modifications, depression during pregnancy?
**Findings:** Adjusting for potential confounders, the risk of discontinuing antidepressants during pregnancy was nearly four times higher in slow metabolizers compared to fast metabolisers. CYP2D6 status did not impact dosage modifications. Twenty percent of women using antidepressants remained depressed.
**Meaning:** Prior knowledge of *CYP2D6* genotype may help to identify pregnant women at greater risk of antidepressant discontinuation. Usage of antidepressants does not necessarily adequately treat maternal depression during pregnancy, suggesting the need for personalized treatment of depression during pregnancy.

## Introduction

Antidepressants are among the most frequently prescribed medications during pregnancy. Up to 10% of women use antidepressants at some point in time during their pregnancy (Cooper et al., 2007), and this rate has been increasing continuously over the past 20 years (Wichman et al., 2008; Dawson et al., 2016). Up to half of pregnant women discontinue antidepressant treatment within the first 6 weeks of gestation and safety concerns may be one of a number of reasons for the discontinuation (Petersen et al., 2011). The discontinuation of antidepressant may cause the re-emergence of the primary psychiatric disorder in pregnant women with severe depression (Rosenbaum and Zajecka, 1997; Nonacs and Cohen, 2003).

Many antidepressants are metabolized via the cytochrome P450 2D6 (CYP2D6) pathway (Kirchheiner et al., 2004), and the activity of this enzyme varies markedly among individuals from poor to ultrarapid metabolism on the basis of the polymorphism of the *CYP2D6* gene (Thuerauf and Lunkenheimer, 2006). Differences in CYP2D6 activity of individuals can affect plasma concentrations of antidepressants, and thus determine the efficacy of the treatment and susceptibility to adverse events (Grasmader et al., 2004). In addition, pregnancy itself can affect *CYP2D6* activity with profound variations in the predicted CYP2D6 phenotype, as determined by its genotype (Anderson, 2005; Ververs et al., 2009), which may require changes in dosage to maintain therapeutic antidepressant plasma levels (Lind et al., 2003; Tracy et al., 2005). Failure to make appropriate changes in dosage can result in sub-therapeutic plasma levels and no improvement of depressive symptoms (Tracy et al., 2005).

The ability to predict individual phenotypes and variation in metabolism based on genetic disposition provides the opportunity to bring precision medicine into clinical practice. The Food and Drug Administration (FDA) recommends, but does not require, genetic testing prior to initiating treatment with many selective serotonin reuptake inhibitors (SSRIs) (Berard and Lacasse, 2009), the most commonly dispensed class of antidepressants at present (Ramos et al., 2007). Currently there are no studies investigating the link between antidepressant discontinuation and *CYP2D6* genotype or activity during pregnancy. Hence, we aim to investigate the association between *CYP2D6* genotype, predicted phenotypes (metabolizer status), and the risk of antidepressant discontinuation, dosage modification, and maternal depression during pregnancy.

## Methods

### Study population

**Figure 1:**
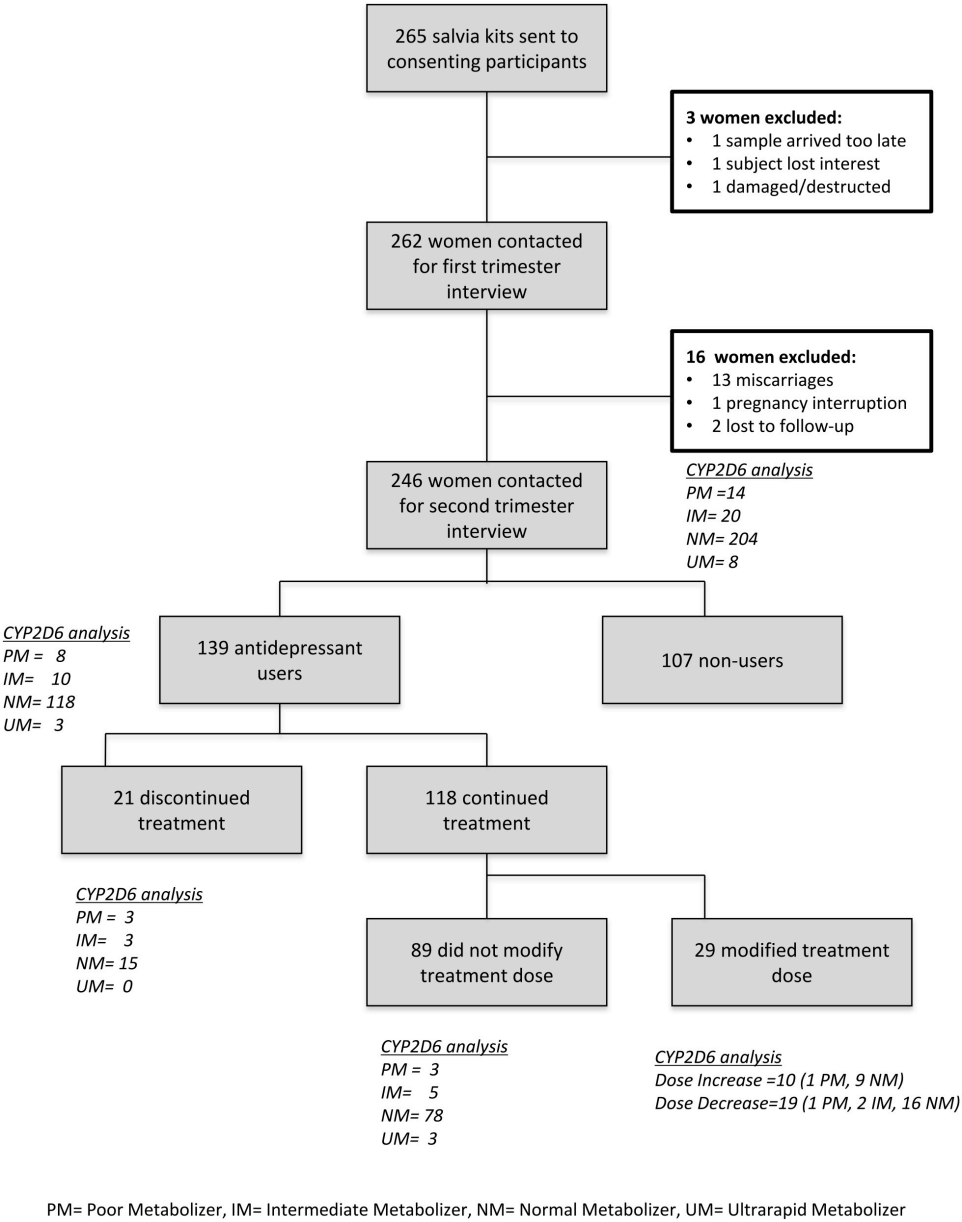
Patient recruitment flow-chart and CYP2D6 predicted phenotypes.

This study was conducted within a subgroup of women sampled within the Organization of Teratology Information Specialists (OTIS) Antidepressants in Pregnancy Cohort. Details of this cohort have been described elsewhere (Karam et al., 2012). Briefly, between 2006 and 2010, pregnant women (within 14 completed weeks of gestation) were recruited from those calling participating OTIS counseling services throughout the United States (US) and Canada [(a) US - Texas Teratology Information Service (TIS); Pregnancy Riskline Utah; New York TIS; Arizona TIS; California TIS; Connecticut Pregnancy Exposure Information Service; and Illinois TIS; and (b) in Canada—Info-Médicaments en Allaitement et Grossesse (IMAGe), CHU Sainte-Justine, Montreal, Quebec; and Motherisk, Hospital for Sick Children, Toronto, Ontario] with questions about medications (mainly antidepressants) exposure in pregnancy, either independently or through their health care providers. Teratogen information services provide free and confidential information to women or their healthcare providers seeking information regarding the risks/benefits associated with taking medications or being exposed to chemicals while being pregnant, planning a pregnancy, or breastfeeding) Recruitment was also conducted via the OTIS website and in the Obstetrics and Gynecology Clinic of CHU Sainte-Justine. Women were eligible at the time of their call to a participating TIS or at the time of enrolment at the clinic of CHU Sainte-Justine if they were: (a) at least 18 years of age; (b) within 14 completed weeks of pregnancy, where beginning of pregnancy was defined as the first day of their last menstrual period, self-reported by women; (c) exposed to an antidepressant (for the exposed group) or having any exposure considered non-teratogenic medications (for the non-exposed group); (d) able to read and understand French or English; and (e) provide written informed consent. Women were excluded if they were: (a) exposed to fetotoxic medications other than the study drugs (Table S1; Kulaga et al., 2009); (b) using antidepressants in the 12 months before pregnancy (for the non-exposed group); or (c) exposed to other concomitant psychopharmacological therapies excluding benzodiazepine. A total of 265 pregnant women met inclusion criteria. However, three did not provide saliva samples (loss of interest, sample arrived too late, or damaged/destructed), 14 had an interrupted pregnancy (miscarriage, abortion), and two were lost to follow-up. Finally, 246 pregnant women (93%) were included in this study (Figure 1).

### Data collection

A trained teratology information specialist conducted a telephone interview at the time of subject enrollment (within 14 completed weeks of gestation), and a telephone interviewer-administered questionnaire was used to collect baseline information including: severity of depression, quality of life status, and pregnancy characteristics (pregnancy due date, intended place of delivery, depression status and antidepressant history, co-morbidities, medication and health service utilizations). During the interview, for those who had taken antidepressants before and during pregnancy, they were asked to specify which antidepressants, dosage, when they started, and how long they had been taking it (duration).

After the initial interview, each woman was provided a pregnancy diary in which she was asked to record any additional medication exposures as the pregnancy progressed. This had to include the drug name, dosages, quantities and duration of use. Similar telephone interviews were also performed during the 2nd and 3rd trimesters of pregnancy.

### Antidepressant exposure

Mothers reported data on medication use, duration and dosage during telephone interviews and in their pregnancy diary. We considered use of selective serotonin reuptake inhibitors (SSRIs); serotonin-norepinephrine reuptake inhibitor (SNRIs); tricyclic antidepressants (TCAs); and atypical antidepressants (bupropion, trazodone, mirtazapine). Three comparator groups were defined: (1) continued users throughout pregnancy, (2) discontinued users, and (3) unexposed. Continued users consisted of women who continued antidepressant treatment throughout pregnancy; this included those who switched to another antidepressant or modified dosages. Further stratifications were done on whether dosage remained unchanged, increased or decreased. Discontinued users consisted of those who stopped using antidepressants during pregnancy.

### Measurement of depression and anxiety

The presence of depressive symptoms and anxiety was measured at every trimester using the telephone interviewer-administered Edinburgh Postnatal Depression Scale (EPDS) (Murray and Carothers, 1990), and Beck Anxiety Inventory (BAI) (Creamer et al., 1995), respectively. Details of these scales have been described previously (Karam et al., 2012, 2016). Table S2 summarizes the scale characteristics. Briefly, the EPDS has been validated in pregnant women and used in clinical research (Eberhard-Gran et al., 2001). It includes 10 items, which describe depressive symptoms to generate a total score ranging from 0 to 30. The cut-off score for screening for depression (major and minor depression) is 13 during pregnancy. The BAI consists of 21 items corresponding to symptoms of anxiety, and gives a continuous overall score. Anxiety is categorized in three levels of severity: mild (score of 8–15), moderate (score of 16–25), or severe (score of 26–63).

### Saliva samples

Saliva samples were collected during the first trimester of pregnancy using Oragen (OG 250) saliva kits (DNA Genotek Inc, Ottawa, Ontario, Canada). Saliva collection kits were mailed to each participant for saliva self-sampling; kits were returned to the coordinating center in Montreal, Quebec, Canada, and stored at 4°C until DNA was isolated. DNA was extracted with a QIAamp DNA Mini Kit (Qiagen, Valencia, CA) and integrity verified by agarose gel electrophoresis. DNA concentration was determined spectrophotometrically using a NanoDrop 1000 instrument (Thermo Fisher Scientific, Waltham, MA) and adjusted to 15 ng/μl unless concentrations were less.

### *CYP2D6* genotype analysis

*CYP2D6* genotyping was carried out according to procedures described in detail elsewhere (Brown et al., 2016). Briefly, a 6.6 kb long-range (XL) PCR fragment was amplified with *CYP2D6*-specific primers. Formation of the PCR product was verified by agarose gel electrophoresis before it was diluted and used as a template to detect SNPs and other sequence variations including nucleotide deletions and insertions. TaqMan (Applied Biosystems, Foster City, CA, USA) or RFLP assays were employed for the detection of sequence variations. Genotyping included the following single nucleotide variations (SNVs): 100C>T (rs1065852), 1023C>T (rs28371706), 1707delT (rs5030655), 1846G>A (rs1800716), 1862ins18bp (no rs), 2549delA (rs35742686), 2615delAAG (rs5030656), 2850C>T (rs16947), 2935A>C (rs5030867), 2988G>A (rs28371725), 3183 G>A (rs59421388), 3201C>T (rs147960066; tested only in the presence of a CYP2D6^*^10 allele) and the exon 9 conversion (no rs). The CYP2D6^*^5 gene deletion, the presence of gene duplications/multiplications and *CYP2D7/2D6* hybrid genes were assayed by XL-PCR. An XL-PCR product of approximately 8 kb in length encompassing the duplicated gene was generated and subsequently genotyped (Gaedigk et al., 2007) for selected SNPs to unequivocally determine the allelic variation of gene duplications (to discriminate, for example, between CYP2D6^*^2x2/^*^4 and ^*^2/^*^4x2). Samples positive for a duplications/multiplication were also tested by a quantitative gene copy number variation (CNV) assay to determine the number of gene copies present in each sample (Gaedigk et al., 2012). The following *CYP2D6* allelic variants were determined using the aforementioned tests: CYP2D6^*^2, ^*^3, ^*^4, ^*^5 (gene deletion), ^*^6, ^*^7, ^*^9, ^*^10, ^*^13 (2D6/2D7 hybrid genes), ^*^17, ^*^29, ^*^41, ^*^56 and gene duplications/multiplications. Alleles were assigned according to the Human Cytochrome P450 Nomenclature database at www.cypalleles.ki.se/cyp2d6.htm. Alleles carrying no SNVs were defaulted to CYP2D6^*^1 and those carrying only 2850C>T to CYP2D6^*^2 assignments, respectively. *CYP2D6* genotypes were translated into phenotype according to the guidelines published by the Clinical Pharmacogenetics Implementation Consortium (CPIC) (Hicks et al., 2015). Table S3 summarizes the main active and inactive alleles associated with different phenotypes. Genotyping was done at Children's Mercy Kansas City, Missouri. Ultrarapid metabolizer (UM), normal metabolizer (NM), intermediate metabolize (IM), and poor metabolizer (PM) categories were described according to Gaedigk et al. (2008) Tables S4, S5 summarizes antidepressants metabolism and excretion.

### Statistical analyses

Subject characteristics are presented as means and proportions for continuous and categorical variables, respectively. Among users of antidepressants at the beginning of pregnancy, crude and adjusted odds ratios (ORs) with 95% confidence intervals (95% CI) were calculated using univariate and multivariate logistic regression models to quantify the association between predicted CYP2D6 phenotype groups and the risk of antidepressant discontinuation, dosage modification, and the occurrence of maternal depression during pregnancy. Severity of depression, maternal age, marital status (living alone or cohabiting), education level (≤12 or >12 years), race (Caucasian vs. others), body mass index (BMI, kg/m^2^), vitamin supplement intake, and smoking status were considered as potential confounders if crude estimates resulted in *p*-values of less than 0.10. “Duration of antidepressant use” and “reasons for discontinuation” were not considered in the analyses given that they are intermediate variables in the causal pathway between the exposure and the outcome (Rau et al., 2004; Mulder et al., 2005; Bijl et al., 2008), and not sharing a common cause with *CYP2D6* genotype (Shenfield, 2004; D'Empaire et al., 2011).

Sensitivity analyses were also performed excluding pregnant women on bupropion monotherapy given that CYP2D6 is a minor pathway for this medication. In addition, given that the CPIC guidelines (Hicks et al., 2015) indicate that paroxetine and fluvoxamine inhibit *CYP2D6*, and thus treatment adjustments are not warranted based on CYP2D6 status for those who are *CYP2D6* normal or intermediate metabolizers, further analyses were done also excluding pregnant women on paroxetine or fluvoxamine. All statistical analyses were performed using SAS (Version 9.02). In this study, the collection of antidepressant usage data was carried blindly to the pharmacogenetic status.

### Ethics

This study was approved by CHU Sainte-Justine's Ethics Committee. This human subjects review and approval served to meet necessary human subjects concerns at all sites. All participants signed an informed consent form.

## Results

Among the 246 participants, the majority (*n* = 204, 83%) had *CYP2D6* genotypes predicting normal metabolism, 3.3% (*n* = 8) were predicted to have ultrarapid metabolism; 5.7% (*n* = 14) poor, and 8.1% (*n* = 20) intermediate metabolism. Pregnant women were on average 31 years old, and at 10 weeks of gestation at the time of recruitment (Table 1).

**Table 1:** Characteristics of participants at enrollment (1st trimester of pregnancy), according to *CYP2D6* predicted phenotypes.

			**Genotype-predicted phenotype**							
	**TOTAL (*n* = 246)**		**PM (*n* = 14; 5.7%)**		**IM (*n* = 20; 8.1%)**		**NM (*n* = 204; 82.9%)**		**UM (*n* = 8; 3.3%)**	
Age–year (mean, SD)	31.2	4.2	31.8	4.0	30.4	4.2	31.3	4.2	30.8	5.3
Caucasian-no. %	225	91.5	14	100.0	17	85.0	188	92.2	6	75.0
Gestational age at enrolment: week (mean, SD)	10.2	3.2	9.9	3.3	10.6	2.7	10.2	3.3	11.4	2.1
BMI prior to pregnancy (kg/m^2^) (mean, SD)^*^	25.0	5.9	28.5	7.6	24.8	7.9	24.8	5.6	23.2	3.3
Post-secondary education-no.%^*^	209	85.0	10	71.4	17	85.0	176	86.3	6	75.0
**SMOKING-NO.%**										
Prior to pregnancy^*^	55	22.4	5	35.7	4	20.0	43	21.1	3	37.5
During the first trimester^*^	22	8.9	0	0.0	1	5.0	19	9.3	2	25.0
**USED VITAMINS-NO.%**										
Prior to pregnancy	147	59.8	6	42.9	12	60.0	127	62.3	2	25.0
During the first trimester	237	96.3	14	100	20	100	196	96.1	7	87.5
**MARITAL STATUS**										
Living alone-no. %	7	2.9	1	7.1	2	10.0	4	2.0	0	0.0
**ANTIDEPRESSANT DRUGS-NO.%**										
Exposed to antidepressants-no.%^*^	139	56.5	8	57.1	10	50.0	118	57.8	3	37.5
0	107	43.5	6	42.9	10	50.0	86	42.2	5	62.5
1	119	48.4	7	50.0	8	40.0	102	50.0	2	25.0
2	20	8.1	1	7.1	2	10.0	16	7.8	1	12.5
**CLASS OF ANTIDEPRESSANTS USED-NO.%**										
SSRI	71	51.1	6	75.0	5	50.0	58	49.2	2	66.7
SNRI	39	28.1	1	12.5	2	20.0	36	30.5	0	0.0
TCA	1	0.7	0	0.0	0	0.0	1	0.8	0	0.0
Other AD	28	20.1	1	12.5	3	30.0	23	19.5	1	33.3

*UM, ultrarapid metabolizer; NM, normal metabolizer; IM, intermediate metabolize; PM, poor metabolizer; SD, standard-deviation; BMI, body mass index; SSRI, Selective Serotonin Reuptake Inhibitor; SNRI, Serotonin-Norepinephrine Reuptake Inhibitors; TCA, Tricyclic Antidepressants; AD, antidepressants*.

* *p < 0.05*.

More than half of the participants (*n* = 139, 57%) were taking antidepressants at recruitment. Specifically, the majority of antidepressant users had a diagnosis of depression/anxiety before pregnancy, including 28.5% major depression, 11.1% mild/moderate depression, 9.6% situational depression, 31.5% general anxiety disorder, 8.9% other anxiety disorders (World Health Organization, 1978, 2010) while 16.3% of depression diagnoses at recruitment remain unknown. The mean duration of antidepressant exposure before pregnancy was 33.9 ± 35.8 months. Discontinuation or dosage modification was reported during the 1st and/or 2nd trimesters. The majority of those treated with antidepressants for depression/anxiety were on monotherapy (*n* = 119, 86%), with a small number on combination therapy (*n* = 20, 14%) (Table S6). SSRI as a class was the most used (51%). However, venlafaxine (27%) was the most utilized followed by citalopram (18%), sertraline (12%), paroxetine (7%), and fluoxetine (7%). The most frequently used combination was SSRI and bupropion. All poor metabolizers were Caucasian, and there was a lower proportion of Caucasians amongst the ultrarapid metabolizers (75%, *n* = 6). Poor metabolizers tended to have a higher pre-pregnancy body mass index (BMI) compared to pregnant women with other phenotypes, with a mean pre-pregnancy BMI of 28.5 kg/m. A higher proportion of poor metabolizers (*n* = 5; 35.7%) and ultrarapid metabolizers (*n* = 3; 37.5%) were smokers compared to normal (*n* = 43; 21.2%) or intermediate (*n* = 4; 20%) metabolizers prior to pregnancy; however, all poor metabolizers and more than half of the total number of women stopped smoking in the first trimester. Nearly 60% of women were using vitamins before pregnancy; this increased to 96.3% once the pregnancy was diagnosed.

Table 2 presents depression and anxiety status according to CYP2D6 phenotype. A third of the women (44/139, 32%) displayed depressive symptoms in the first trimester of pregnancy (mean EPDS score ≥13), and all of them had anxiety (96.7% low, and 3.3% moderate to severe anxiety).

**Table 2:** Antidepressant usage/dosage modification, and maternal depression and anxiety, according to *CYP2D6* predicted phenotype.

	**Genotype-predicted phenotype**									
	**TOTAL (*n* = 246)**		**PM (*n* = 14; 5.7%)**		**IM (*n* = 20; 8.1%)**		**NM (*n* = 204; 82.9%)**		**UM (*n* = 8; 3.3%)**	
**FIRST TRIMESTER**										
Maternal depression (EPDS score)^*^	4.9	4.9	5.6	4.3	2.1	2.0	5.2	5.1	4.0	4.0
Median (min-max)-no.%	3	0–21	4.5	0–14	1.5	0–7	4	0–21	3	0–11
Depressive women (score 13+)-no.%^*^	44	18.0	2	14.3	0	0.0	41	20.2	1	12.5
Maternal anxiety (BAI score)	6.4	6.1	8.0	5.6	4.9	4.5	6.5	6.3	4.8	5.1
Median (min-max)-no.%	4	0–36	6	3–20	4	0–19	4	0–36	3	1–15
Low anxiety-no.%	237	96.7	14	100.0	20	100.0	158	95.8	8	100.0
Moderate to severe anxiety-no.%	8	3.3	0	0.0	0	0.0	8	3.9	0	0.0
**SECOND TRIMESTER**										
AD dosage modification during pregnancy no.%	*n* = 139		*n* = 8		*n* = 10		*n* = 118		*n* = 3	
No modification	89	64.0	3	37.5	5	50.0	78	66.1	3	100.0
Discontinuation	21	15.1	3	37.5	3	30.0	15	12.7	0	0.0
Increase in dosage	10	7.2	1	12.5	0	0.0	9	7.6	0	0.0
Decrease in dosage	19	13.7	1	12.5	2	20.0	16	13.6	0	0.0

*Plus–minus values are means ± standard deviation (SD), unless otherwise stated*.

*UM, ultrarapid metabolizer; NM, normal metabolizer; IM, intermediate metabolize; PM, poor metabolizer; EPDS, Edinburgh Postnatal Depression Scale; BAI, Beck Anxiety Inventory*.

* *p < 0.05*.

**Figure 2:**
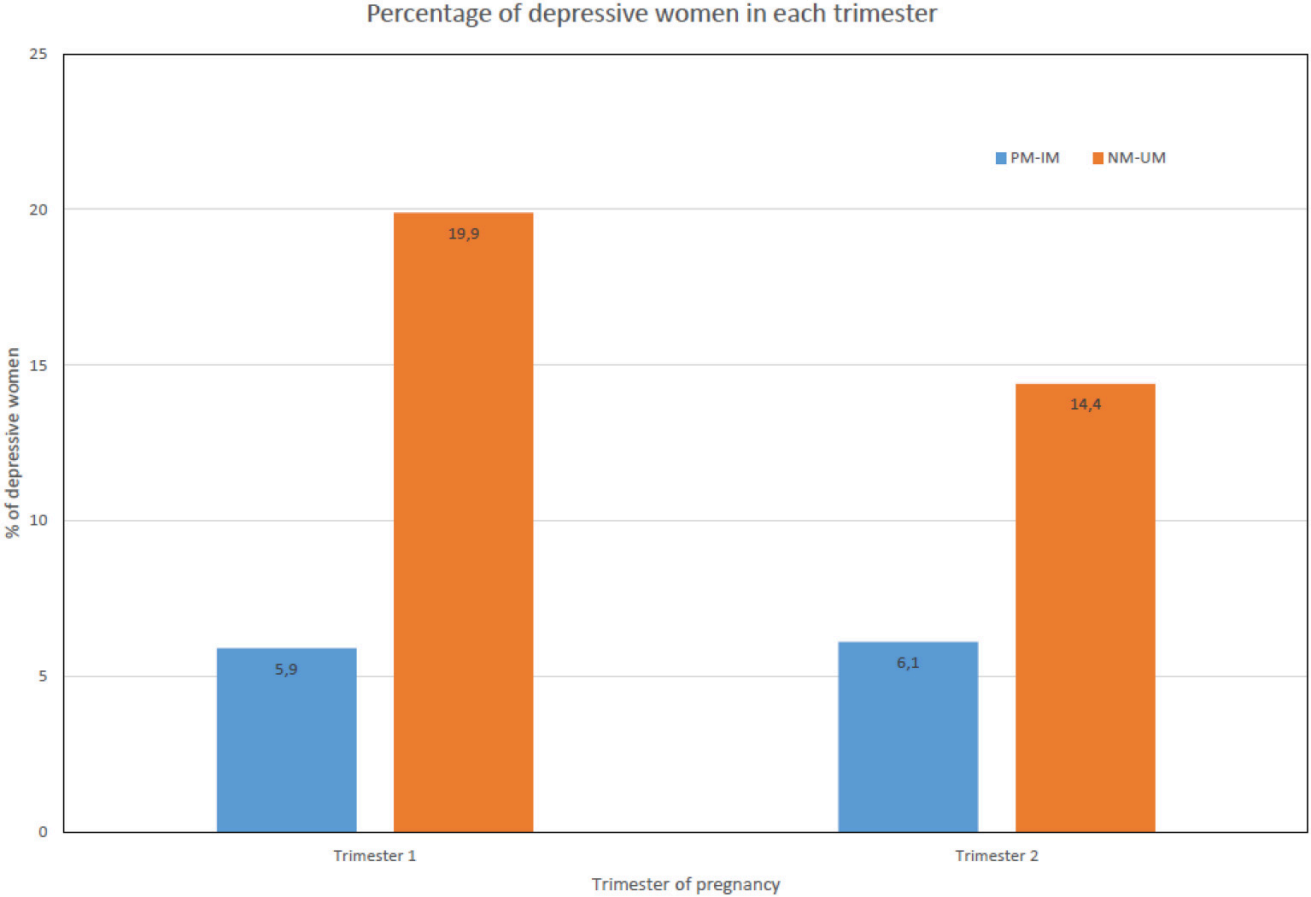
Proportion of women with depressive symptoms during pregnancy. UM, Ultrarapid metabolizers; NM, normal metabolizers; IM, intermediate metabolizers; PM, poor metabolizers.

Overall, the majority of pregnant women using antidepressants did not modify their dosage during gestation (89/139, 64%); 13.7% (19/139) decreased, 7.2% (10/139) increased their dosage, and 15% (21/139) discontinued treatment (Table 2) which all occurred in the first trimester. Compared with slow metabolizers, significantly higher proportion of women in the faster metabolizer group had depressive symptom in the first trimester (19.81 vs. 5.88%, *p* = 0.049). The proportion of women with depressive symptom in the second trimester was also higher among the faster metabolizer group compared to slow metabolizers but the difference was not statistically significant (14.35 vs. 6.06%, *p* = 0.19; Figure 2).

Adjusting for depressive symptoms, and other potential confounders, the risk of discontinuing antidepressants during pregnancy was nearly four times higher in slow metabolizers (poor or intermediate metabolizers) compared to those with a faster metabolism rate (normal or ultrarapid metabolizers), ORadj = 3.57 (95% CI: 1.15–11.11) (Table 3). *CYP2D6* genotype-predicted phenotype had no statistically significant impact on whether antidepressant dosage was increased or decreased (Table 4).

**Table 3:** Genotype-predicted CYP2D6 phenotype and the risk of antidepressant discontinuation during pregnancy.

**Variables**	**Crude OR**	**95% CI**	**Adjusted OR**	**95% CI**
*CYP2D6* predicted phenotype (NM-UM vs. PM-IM)	4.55	1.32–14.29	3.57	1.15–11.11
Smokers (yes/no)	0.68	0.26–1.75	0.50	0.17–1.45
Post-secondary education (yes/no)	0.69	0.23–2.10	0.71	0.21–2.48
Obesity (BMI ≥ 30 kg/m^2^)	1.63	0.53–4.98	1.49	0.45–4.91
EPDS score (≥13 vs. <13)	1.03	0.37–2.88	1.30	0.43–3.95

*UM, ultrarapid metabolizer; NM, normal metabolizer; IM, intermediate metabolize; PM, poor metabolizer; BMI, body mass index; EPDS, Edinburgh Postnatal Depression Scale; OR, odds ratio; 95%CI, 95% confidence interval*.

**Table 4:** Genotype-predicted CYP2D6 phenotype and the risk of antidepressant dosage modification during pregnancy.

**Variables**	**Crude OR**	**95% CI**	**Adjusted OR**	**95% CI**
*CYP2D6* predicted phenotype (NM-UM vs. PM-IM)	1.10	0.33–3.57	1.02	0.29–3.57
Smoking (yes/no)	0.89	0.39–2.03	0.97	0.41–2.30
Post-secondary education (yes/no)	0.66	0.25–1.76	0.65	0.23–1.84
Obesity (BMI ≥ 30 kg/m^2^)	1.00	0.34–2.95	0.93	0.31–2.81
EPDS score (≥13 vs. <13)	0.97	0.39–2.42	0.93	0.36–2.40

*UM, ultrarapid metabolizer; NM, normal metabolizer; IM, intermediate metabolize; PM, poor metabolizer; BMI, body mass index; EPDS, Edinburgh Postnatal Depression Scale; OR, odds ratio; 95%CI, 95% confidence interval*.

Sensitivity analyses were performed excluding pregnant women on bupropion monotherapy. All bupropion monotherapy users were normal metabolizers (*n* = 5; Table S3), and excluding them from analyses did not significantly change results [ORadj = 3.42 (95% CI: 1.29–9.07)]. Further excluding pregnant women on paroxetine or fluvoxamine did not statistically change findings [ORadj = 3.07 (95% CI: 1.06–8.89)]. However, the association between genotype-predicted CYP2D6 phenotype and paroxetine or fluvoxamine discontinuation during pregnancy was not statistically significant [ORadj = 1.87 (95% CI: 0.89–3.93)].

## Discussion

To our knowledge, this is the first study to investigate the effect of *CYP2D6* genotype on the risk of antidepressant discontinuation, dosage modification, and maternal depression during pregnancy. We found that pregnant women with a poor or intermediate phenotype were more likely to discontinue their antidepressants during pregnancy (by approximately 4-fold) compared to pregnant women with normal or ultrarapid phenotypes.

Pregnancy accentuates the differences in plasma concentration of antidepressants between the slow metabolizers (poor and intermediate metabolizers) and fast metabolizers (normal and ultrarapid metabolizers), as the CYP2D6 activity increases during pregnancy (Tracy et al., 2005) in fast metabolizers but not in poor metabolizers (Anderson, 2005; Ververs et al., 2009). Consequently, during pregnancy the plasma concentrations of CYP2D6-dependent antidepressants was decreased in fast metabolizers but increased in poor metabolizers, as poor metabolizers do not express functional *CYP2D6* and intermediate metabolizers only possess one decreased function allele. Thus the metabolism of CYP2D6-dependent antidepressants depends on other enzymes, which may have decreasing activity during pregnancy (Ververs et al., 2009; Anderson, 2005). Thus, our findings are specifically related to the pregnancy period, and further study is warranted to extrapolated to other periods in an individual's life.

Our findings are consistent with the literature. Several pharmacogenetic studies on antidepressant induced side effects (mouth dryness, nausea, and restlessness) reported that CPY2D6 poor metabolizers had an increased frequency and severity of concentration-dependent side effects due to slow elimination (Rau et al., 2004; D'Empaire et al., 2011). A retrospective follow-up study of depressed inpatients found that CYP2D6 poor metabolizers had more frequent switches (RR 3.50, 95% CI 1.52-8.10), and more dosage modifications (RR 2.18, 95% CI 1.36-2.95), which was indicative of an overall expression of unsatisfactory response to treatment including both treatment failure and unacceptable side effects (Mulder et al., 2005).

Factors predicting adherence and persistence are complex and interactive (Hung, 2014). More than one third of women who continue medications throughout pregnancy frequently consider discontinuing (Mulder et al., 2012). As such, we hypothesize that the decision to continue or discontinue antidepressants are multi-factorial for each pregnant woman. Safety concerns with regard to taking antidepressants during pregnancy is one reason for discontinuation. In fact, pregnant women were found to be more likely to discontinue antidepressants than non-pregnant women, particularly in the first 6 weeks of pregnancy (Petersen et al., 2011).

In addition, concentration-dependent side effects are another important factor predicting poor adherence and discontinuation of antidepressants (Ferguson, 2001; De las Cuevas et al., 2014). Increased plasma antidepressant concentration has been seen in slow metabolizers (Ververs et al., 2009), which would in turn lead to an increased frequency and severity of concentration-dependent adverse drug effects (Ferguson, 2001; Rau et al., 2004). For this reason, it remains a challenge to continue the use of antidepressants for women who have already experienced adverse drug effects from these drugs, as they have experienced worse morning sickness than non-users (Bozzo et al., 2011). These adverse drug reactions caused by antidepressants may become unbearable, especially in slow metabolizers (poor and intermediate metabolizers), leading them to discontinue the use of the drug (Misri et al., 2013).

In addition, in our study of 246 pregnant women, genotype testing found that 3.3% (*n* = 8) had *CYP2D6* genotypes predicting ultrarapid metabolism, 8.1% (*n* = 20) intermediate metabolism, and 5.7% (*n* = 14) poor metabolism. The genotype distribution of the *CYP2D6* genotype frequencies and predicted phenotypes were within the reported ranges for Caucasians (Sanchez-Iglesias et al., 2016). Previous studies indicated a higher prevalence of poor metabolizers (ranging from 5 to 10%; Bertilsson et al., 1997) amongst Caucasians compared to other ethnicities. In our study, all poor metabolizers were Caucasian, which is consistent with the majority of our study population being Caucasian.

In the present study, one third of antidepressant users had depressive or anxiety symptoms in the first trimester (44/139), and 21% of women exposed to antidepressants throughout pregnancy remained depressed. These data are comparable with previous studies. Cohen and colleagues reported that 26% (21/82) of women remained depressed while maintaining their antidepressant treatment throughout their pregnancy (Cohen et al., 2006). Swanson et al. reported that 16.9% (743/4,390) of women with depression had been hospitalized for depression while continuing using antidepressants during pregnancy (Swanson et al., 2015). Marcus et al. reported that 52% (36/68) of those taking antidepressant medication showed depressive symptoms (Marcus et al., 2005). Our data also show that higher proportion of women in the fast metabolizer group had depressive symptoms in comparison with the slow metabolizer group in the first and second trimester of pregnancy (Figure 2). A previous study has also reported that the proportion of women with depressive symptoms was significantly increased during the course of pregnancy in the fast metabolizers group but not in the slow metabolizer group (Ververs et al., 2009). These data indicate that better strategies for the management of depression during pregnancy, such as personalized drug treatment using therapeutic monitoring during pregnancy to achieve optimal and safe response, are urgently needed (DeVane et al., 2006). The FDA already recommends genetic testing prior to initiating treatment with many SSRIs (Berard and Lacasse, 2009). Genotyping before treatment has been shown to be useful for drugs with a narrow therapeutic index, or for patients on multiple medications (Dahl and Sjoqvist, 2000). Genetic biomarkers can help identify patients at increased risk of antidepressant treatment failure and/or adverse side effects. Pre-emptive *CYP2D6* genotyping allows the clinician to adjust dosage appropriately or use different treatment options. We are aware that citalopram, escitalopram, and sertraline are not exclusively metabolized by CYP2D6 (Tsai et al., 2010; Drozda et al., 2014), and these three products represent 50 out of 119 subjects in the monotherapy group of our study. Nevertheless, further work is warranted to standardize genotyping, translate genotype data into phenotype, and develop guidelines to optimize drug therapy during pregnancy (Kalman et al., 2016).

In this study, 20 women used two antidepressants, and bupropion is the most frequently used co-antidepressant (*n* = 10). Bupropion is metabolized in the liver by CYP2B6 (Jefferson et al., 2005), and it is also a CYP2D6 inhibitor (D'Empaire et al., 2011), which could potentially lead to an interaction at the level of this enzyme of interest (D'Empaire et al., 2011). Co-medication with a potent CYP2D6-inhibitor can convert patients with fast metabolizers genotypes into poor metabolizer phenotypes (Gressier et al., 2015; Sanchez-Iglesias et al., 2016). Thus, for fast metabolizers, co-administrating of bupropion with other CYP2D6-dependent antidepressants increases the plasma concentration of the CYP2D6-dependent antidepressants (Preskorn, 2003; Sanchez-Iglesias et al., 2016). However, since slow metabolizers (poor and intermediate metabolizers) inherit two no function alleles which leads to absent CYP2D6 activity, co-administrating a CYP2D6-inhibitor in CYP2D6 poor metabolizers will lead to a decreased/absent CYP2D6 activity at most (Lessard et al., 1999). Thus, the potential interaction caused by the inclusion of bupropion for example could lead to a decrease in the difference between the two comparison groups and therefore underestimate the association between CYP2D6 activity and antidepressant discontinuation, leading to conservative findings.

In this study, the participants were recruited from women calling participating OTIS counseling services throughout the United States and Canada with questions about medications (mainly antidepressants) exposure in pregnancy, either independently or through their health care providers (e.g., nurses, midwives, physicians, and pharmacists). Recruitment was also conducted via the OTIS website and in the Obstetrics and Gynecology Clinic of CHU Sainte-Justine (Montreal, Quebec, Canada). In North America, antidepressants are prescription-only medication, and prescribed either by a general practitioner or by a psychiatrist, similar as in other countries. However, during the pregnancy period, women's obstetrician may also be involved in the management of their medications. As such, approximately 57% (*n* = 139) of pregnant women were taking antidepressants at recruitment. This rate is much higher than findings from the Quebec Pregnancy Cohort (Berard and Sheehy, 2014) or from the Swedish Birth Register (Kallen et al., 2011) for example, where the prevalence rate of antidepressant use before and during pregnancy is 4.5% in both studies. This can be partly explained by the eligibility criteria for this study, and the underlying populations that are different in our study (TIS callers) compared to population-based studies such as the Quebec Pregnancy Cohort and Swedish Birth Register. Although gestational antidepressant usage was self-reported, it is unlikely to be subjected to recall bias because it was collected in real-time during each trimester of pregnancy, before delivery. We do not know why pregnant women discontinued or modified dosage. Other limitations include lack of statistical power in analyses on dosage modifications; especially given the small sample size in those with predicted poor or intermediate phenotypes. Finally, given the number of comparisons made, chance findings cannot be ruled out.

The prospective design, and centralized follow-up including pregnant women in nine participating teratology information services in the US and Canada is the major strength of this study. Information from patients was collected in real-time using standardized questionnaires and instruments at various stages during pregnancy, which minimized potential recall bias. In addition, data included various determinants of antidepressant discontinuation during pregnancies, which were used for adjustments in the analyses (Grzeskowiak et al., 2011). Well-established and validated instruments such as the EPDS were used to measure depressive symptoms (Murray and Carothers, 1990; Eberhard-Gran et al., 2001). Data collection and analyses were centralized in Montreal, Quebec, Canada, and genotyping was performed at Children's Mercy Kansas City, MO, using validated methods, increasing the reliability of the results.

The biological plausibility behind the findings of this study is that the CYP2D6 activity increases during pregnancy (Tracy et al., 2005) in fast metabolizers (normal and ultrarapid metabolizers), but not in slow metabolizers (poor and intermediate metabolizers) (Anderson, 2005; Ververs et al., 2009). Since the CYP2D6 activity increase is ~25% in the first trimester, ~35% in the second trimester and ~50% in the third trimester as compared with the postpartum period (Tracy et al., 2005), our findings would also apply to the time of delivery as well as the acute post-partum phase. However, further investigations could be conducted to confirm this statement. In addition, our study supports previous findings that a higher proportion of women in the fast metabolizer group had depressive symptoms during the course of pregnancy compared with women in the slow metabolizer group (Ververs et al., 2009). A previous study found that the frequency of CYP2D6 ultrarapid metabolizers among women being depressed both during late pregnancy and postpartum was around six times higher than expected in a general population, suggesting ultrarapid metabolizers are more vulnerable to the vast changes of metabolism in the time around parturition (Josefsson et al., 2004). Depression during pregnancy and especially postpartum might be particularly hazardous as it affects not only the women but also the mother-infant interaction. The putative relationship between *CYP2D6* genotype and depression symptoms in late pregnancy and/or after delivery warrants further investigation.

## Conclusions

For pregnant women who are treated with antidepressants, those with poor or intermediate metabolic phenotypes are at a higher risk of discontinuing therapy during pregnancy. In addition, approximately 21% of treated women remained depressed during pregnancy (14.4% NM-UM; 6.1% PM-IM), indicating an urgent need for personalized treatment of depression during this critical time-period.

## Author contributions

AB had full access to all of the data in the study and takes responsibility for the integrity of the data and the accuracy of the data. Analyses: AB, AG, OS, CC, MR, PB, DJ, KK, SL, LW, DQ, KD, and JZ. Study concept and design: AB, AG, OS, and JP. Acquisition of data: All. Measurement of depression and anxiety: AB and OS. Genotype analysis: AG. Analysis and Interpretation of data: All. Drafting of the manuscript: JZ and AB. All authors revised the manuscript for important intellectual content. All authors gave final approval of the final version for the submission.

### Conflict of interest statement

All authors have completed the ICMJE uniform disclosure form at http://www.icmje.org/coi_disclosure.pdf and declare: AB is a consultant for plaintiffs in litigations involving antidepressants and birth defects. The reviewer SL and handling Editor declared their shared affiliation, and the handling Editor states that the process nevertheless met the standards of a fair and objective review.
