# Supplementary material for: Association between CYP2D6 Genotypes and the Risk of Antidepressant Discontinuation, Dosage Modification and the Occurrence of Maternal Depression during Pregnancy
Source: Front Pharmacol. 2017 Jul 17;8:402. doi: 10.3389/fphar.2017.00402 (PMC5511844; doi:10.3389/fphar.2017.00402)
Supplement: Supplementary file 1 [file Table1.PDF]

Supplementary Appendix

This appendix has been provided by the authors to give readers additional information about their work.

Supplement to: *CYP2D6* genotypes and the risks of antidepressant discontinuation, dosage modification and the occurrence of maternal depression during pregnancy. Anick Bérard, Andrea Gaedigk, Odile Sheehy, Christina Chambers, Mark Roth, Pina Bozzo, Diana Johnson, Kelly Kao, Sharon Lavigne, Lori Wolfe, Dee Quinn, Kristen Dieter, Jin-Ping Zhao, and the OTIS (MotherToBaby) Collaborative Research Group

Contents:

Table S1. List of prescribed drugs with fetotoxic potential in human - updated April 2014

Table S2. Scales used to assess depression and anxiety

Table S3. Common allele descriptions used to classify different *CYP2D6* phenotypes

Table S4. *CYP2D6* enzymes involved in biotransformation of SSRIs

Table S5. Antidepressants metabolism and excretion

Table S6. Antidepressants used according to *CYP2D6* predicted phenotype

Table S1. List of prescribed drugs with fetotoxic potential in human - updated April 2014

Class	Denomination commune	Code
**Systemic retinoids**	isotretinoin	43319
	acitretin	46237-47101
	vitamin A	10114
	vitamin A, D and C	10192
	mulivitamin A, D and C	47242
**Antiepileptics**	carbamazepin	1404
	phenytoin	43462-43449
	phenobarbitol	17906-7345
	primidone	8047
	valproic acide	38951
	divalproex sodique	44073
	gabapentine	47100
	lamotrigine	46248-47110
	levetiracetam	47463
	pregabaline	47571
	topiramte	46359-47229
	valproate sodique	39393
	vigabatrin	46477-47080
**Anti-thyroid drugs**	methimazole	40836
	propylthiouracil	8242
**Anti-coagulants**		10205-10218-
	warfarine	46604-47390
	nicoumalone	47653
**Tetracycline derivative**	doxycyclin	3211-3224
	minocyclin	6279
	streptomycyn	9139
	tetracycline	9386
**Angiotensin converting enzyme (ACE) inhibitors**	benazepril	47049
	captopril	42071
	cilazapril	46194-47056
	cilazapril/HCT	47320
	enalapril	45476
	enalapril/HCT	45572
	fosinopril	47002
	lisinopril	45576
	lisinopril/HCT	47040
	perindopril	46258-47117
	perindopril/indapamine	47449
	quinalapril	45629
	quinalapril/HCT	47301
	ramipril	46216-47079
	ramipril/HCT	47655
	trandolapril	47250
	trandolapril/verapamil	47440
	verapamil	40550

**Androgens**	danazol	37677
	testosterone	46741-47401
	testosterone (cypionate de)	9334-46551-47414
	testosterone (enanthate de)	9347
	testosterone (undecanoate de)	46155-47013
	testosterone (propionate de)	9360-46442-46540
	methyltestosterone	6214
**Antineoplastic agents**	anastrozole	46316-47166
	bicalutamide	46297-47162
	busereline (acetate de)	45482-46655
	busulfan	1144
	chlorambucil	1716
	cyclophosphamide	2340
	estramustine (phosphate disodique de)	39744
	etoposide	42760
	exemestane	47388
	fluorouracile	4017
	flutamide	44554
	gosereline (acetate de)	45545
	hydrosyuree	41720
	ifosfamide	45508
	interferon alfa-2B	45444
	interferon alfa-2B (sans albumine)	47314
	letrozole	46383
	leuprolide (acetate de)	44632-46632
	melphalan	5564
	mercaptopurine	5733
	methotrexate	338-47651
	nilutamide	47003
	procarbazine	8112
	tamoxifene (citrate de)	38704
	thioguanine	10790
	trétinoine	39003-40407-43319-46000
	triptoréline (pamoate de)	47594
**Others**	amiodarone	45441
	diethylstilbestrol	2795-2808-46696
	dihydroergotamine	2899
	fluconazole	45568-46360
	leflunomide	46649-47362
	lithium (carbonate de)	5330-47237
	lithium (citrate de)	47071
	methotrexate	338-47651
	penicillamine	6994

	cyclophosphamide	2340
	misoprostol	45445
	diclofenac/misoprostol	46154-47059
	quinine	8489
**Statins**	atorvastatine calcique	46355-47232
	amlodipine/atorvastatine calcique	47609
	cerivastatine	46425-47272
	fluvastatine sodique	47083-46240
	lovastatine	45500
	lovastatine/acide nicotinique	47604
	pravastatine sodique	45570-47169
	pravastatine sodique/AAS	47595
	rosuvastatine calcique	46860
	simvastatine	45564-46584
**Angiotensin-receptor blocker (ARB)**	candersartan	46529-47309
	candersartan/HCT	46760-47412
	eprosartan	47389
	eprosartan/HCT	47532-47534
	irbesartan	46459-47282
	irbesartan/HCT	47354
	losartan	46284-46441-47135
	losartan/HCT	47207
	olmesartan	
	olmesartan/HCT	
	telmisartan	46587-47333
	telmisartan/HCT	47413
	valsartan	46418-47259
	valsartan/HCT	47369

TABLE S2. **Scales used to assess depression and anxiety during each trimester of pregnancy**

**Name of scale**	Outcome measures	Description of scales	Measurements
The Edinburgh Postpartum Depression Scale (EPDS)[11]	Depressive symptoms during pregnancy	Lists ten depressive symptoms. Respondents rate each symptom on a scale of 0-3. A total score ranging from 0-30 is made from a sum of all scores.	Considered to have depressive symptoms if:<br>• Score ≥13 during pregnancy<br>• Score ≥10 after delivery
Beck Anxiety Inventory Scale[12]	Anxiety severity	Consists of 21 items corresponding to a symptom. Each symptom has four choices. The values of score for each symptom are summed to give an overall score.	Level of anxiety:<br>• 8-15 Mild<br>• 16-25 Moderate<br>• 26-63 Severe

TABLE S3. **Common allele descriptions used to classify different *CYP2D6* predicted phenotypes[18]**

Phenotype	Allele description
**Ultrarapid metabolizers**	Consists of three or more active alleles
**Normal metabolizers**	Two active alleles:
	Two reduced activity
	One active and one inactive
	One reduced activity and one active
	Two active alleles
	Example of common active alleles: (*1, *22, *9, *10 and *17)
**Intermediate**	Consist of one allele with reduced activity and one inactive allele.
	For example: carriers of one of (*1, *22, *9, *10, and *17) or other more rare active alleles combined with (*3, *4m, *5m, *6, *7, *8) or other more rare completely deficient alleles
**Poor**	Consists of two Inactive alleles
	For example: *3-8, *11-16, *19-21, *38, *40, *42, *4m, *5m, *6, *7, *8

TABLE S4. ***CYP2D6*** **enzymes involved in biotransformation of SSRIs**

SSRI	Enzymes involved in biotransformation
**Citalopram**	*CYP2C19*, *CYP2D6*, *CYP3A4*
**Escitalopram**	*CYP2C19*, *CYP2D6*, *CYP3A4*
**Fluoxetine**	*CYP2D6*, *CYP2C9*, *CYP2C19*, *CYP3A4*
**Fluvoxamine**	*CYP1A2*, *CYP2D6*
**Paroxetine**	*CYP2D6*, *CYP3A4*
**Sertraline**	*CYP2C9*, *CYP2C19*, *CYP2D6*, *CYP3A4*

CYP: cytochrome P450; SSRI: selective serotonin reuptake inhibitors

TABLE S5. Antidepressants metabolism and excretion

Variables	Metabolism	Excretion
Bupropion	Liver, mostly *CYP2B6*, with some contributions from *CYP1A2*, *CYP2A6*, *CYP2C9*, *CYP3A4*, *CYP2E1* and *CYP2C19*	Renal (87%; 0.5% unchanged), Faecal (10%)
Citalopram	Liver, *CYP3A4* and *CYP2C19*	Mostly as unmetabolized citalopram in urine
Clomipramine	Liver, *CYP2D6*	Renal (60%), faeces (32%)
Duloxetine	Liver, *CYP2D6* and *CYP1A2*	70% in urine, 20% in feces
Escitalopram	Liver, *CYP3A4* and *CYP2C19*	Renal
Fluoxetine	Liver, *CYP2D6*	Urine (80%), faeces (15%)
Fluvoxamine	Liver, cytochrome P450 enzymes. Mostly via oxidative demethylation)	Renal (98%; 94% as metabolites, 4% as unchanged drug)
Mirtazapine	Liver, *CYP1A2*, *CYP2D6* and *CYP3A4*	Urine (75%), Faeces (15%)
Paroxetine	Liver, *CYP2D6*	Renal (64%; 2% unchanged and 62% as metabolites), Faecal (36%; <1% unchanged)
Sertraline	Liver, *CYP2B6*	Renal

TABLE S6. Antidepressants used according to *CYP2D6* predicted phenotype

		Genotype-predicted phenotype, n (%)			
	**TOTAL (n=246)**	**PM (n=14; 5.7%)**	**IM (n=20; 8.1%)**	**NM (n=204; 82.9%)**	**UM (n=8; 3.3%)**
Type of antidepressants used:					
**Monotherapy**:					
Bupropion	5 (3.6)	0 (0.0)	0 (0.0)	5 (4.2)	0 (0.0)
Citalopram	25 (18.0)	4 (50.0)	4 (40.0)	16 (13.6)	1 (33.3)
Clomipramine	1 (0.7)	0 (0.0)	0 (0.0)	1 (0.9)	0 (0.0)
Duloxetine	1 (0.7)	0 (0.0)	0 (0.0)	1 (0.9)	0 (0.0)
Escitalopram	9 (6.5)	0 (0.0)	0 (0.0)	9 (7.6)	0 (0.0)
Fluoxetine	10 (7.2)	1 (12.5)	0 (0.0)	9 (7.6)	0 (0.0)
Fluvoxamine	1 (0.7)	1 (12.5)	0 (0.0)	0 (0.0)	0 (0.0)
Mirtazapine	2 (1.4)	0 (0.0)	0 (0.0)	2 (1.7)	0 (0.0)
Paroxetine	10 (7.2)	0 (0.0)	1 (10.0)	8 (6.8)	1 (33.3)
Sertraline	16 (11.5)	0 (0.0)	0 (0.0)	16 (16.6)	0 (0.0)
Trazodone	1 (0.7)	0 (0.0)	1 (10.0)	0 (0.0)	0 (0.0)
Venlafaxine	38 (27.3)	1 (12.5)	2 (20.0)	35 (29.7)	0 (0.0)
**Bitherapy**:					
Citalopram\buproprion	1 (0.7)	0 (0.0)	0 (0.0)	1 (0.9)	0 (0.0)
Citalopram\mirtazapine	1 (0.7)	0 (0.0)	1 (10.0)	0 (0.0)	0 (0.0)
Citalopram\nortriptilyne	1 (0.7)	0 (0.0)	0 (0.0)	1 (0.9)	0 (0.0)
Citalopram\trazodone	1 (0.7)	0 (0.0)	0 (0.0)	1 (0.9)	0 (0.0)
Escitalopram\bupropion	3 (2.2)	0 (0.0)	0 (0.0)	3 (2.5)	0 (0.0)
Escitalopram\duloxetine	1 (0.7)	0 (0.0)	0 (0.0)	1 (0.9)	0 (0.0)
Escitalopram\mirtazapine	1 (0.7)	0 (0.0)	0 (0.0)	1 (0.9)	0 (0.0)
Escitalopram\trazodone	1 (0.7)	0 (0.0)	0 (0.0)	1 (0.9)	0 (0.0)
Fluoxetine\bupropion	3 (2.2)	1 (12.5)	0 (0.0)	2 (1.7)	0 (0.0)
Paroxetine\amitriptyline	1 (0.7)	0 (0.0)	0 (0.0)	1 (0.9)	0 (0.0)
Paroxetine\trazodone	1 (0.7)	0 (0.0)	0 (0.0)	1 (0.9)	0 (0.0)
Sertraline\bupropion	2 (1.4)	0 (0.0)	1 (10.0)	0 (0.0)	1 (33.3)
Sertraline\trazodone	1 (0.7)	0 (0.0)	0 (0.0)	1 (0.9)	0 (0.0)
Venlafaxine\bupropion	1 (0.7)	0 (0.0)	0 (0.0)	1 (0.9)	0 (0.0)
Venlafaxine\mertazapine	1 (0.7)	0 (0.0)	0 (0.0)	1 (0.9)	0 (0.0)
**Class of antidepressants used**:					
SSRI	71 (51.1)	6 (75.0)	5 (50.0)	58 (49.2)	2 (66.7)
SNRI	39 (28.1)	1 (12.5)	2 (20.0)	36 (30.5)	0 (0.0)
TCA	1 (0.7)	0 (0.0)	0 (0.0)	1 (0.9)	0 (0.0)
Atypical Antidepressants					
Bupropion	5 (3.6)	0 (0.0)	0 (0.0)	5 (4.2)	0 (0.0)
Mirtazapine	2 (1.4)	0 (0.0)	0 (0.0)	2 (1.7)	0 (0.0)
Trazodone	1 (0.7)	0 (0.0)	1 (10.0)	0 (0.0)	0 (0.0)
**Dual classes/antidepressants used:**					
SSRI\SNRI	1 (0.7)	0 (0.0)	0 (0.0)	1 (0.9)	0 (0.0)
SSRI\TCA	2 (1.4)	0 (0.0)	0 (0.0)	2 (1.7)	0 (0.0)
SSRI\Bupropion	9 (6.5)	1 (12.5)	1 (10.0)	6 (5.1)	1 (33.3)
SSRI\Mirtazapine	2 (1.4)	0 (0.0)	1 (10.0)	1 (0.9)	0 (0.0)
SSRI\Trazodone	4 (2.9)	0 (0.0)	0 (0.0)	4 (3.4)	0 (0.0)
SNRI\Bupropion	1 (0.7)	0 (0.0)	0 (0.0)	1 (0.9)	0 (0.0)
SNRI\Mirtazapine	1 (0.7)	0 (0.0)	0 (0.0)	1 (0.9)	0 (0.0)

UM: ultrarapid metabolizer; NM: normal metabolizer; IM: intermediate metabolize; PM: poor metabolizer; SSRI, Selective serotonin reuptake inhibitor; SNRI, Serotonin-norepinephrine reuptake inhibitors; TCA, Tricyclic antidepressants.
